# Community-Acquired Kodamaea ohmeri Fungemia in an Immunocompetent Host Coexisting With Severe Leptospirosis: A Case Report and Review of Community-Acquired Fungemia Cases

**DOI:** 10.7759/cureus.115215

**Published:** 2026-08-26

**Authors:** Sumedh U Jajoo, Ishan Verma, Kusum Saini, Amol Dube, Pawan Kumar Darivemula, Jignesh C Baraiya, Neeta Gade

**Affiliations:** 1 General Medicine, All India Institute of Medical Sciences, Nagpur, Nagpur, IND; 2 Microbiology, All India Institute of Medical Sciences, Nagpur, Nagpur, IND

**Keywords:** community-acquired fungemia, emerging yeast pathogen, immunocompetent host, kodamaea ohmeri, leptospirosis, liposomal amphotericin b

## Abstract

Community-acquired fungemia in immunocompetent hosts remains exceptionally rare. *Kodamaea ohmeri* is an emerging opportunistic yeast pathogen increasingly recognised as a cause of invasive fungal infections, particularly among immunocompromised individuals and in healthcare settings.

A 33-year-old previously healthy female farmer presented with severe leptospirosis with multiorgan dysfunction. Blood cultures obtained before central venous catheter insertion grew *K. ohmeri*, identified by the VITEK 2 YST card and matrix-assisted laser desorption ionisation-time of flight (MALDI-TOF) mass spectrometry. Repeat blood cultures also yielded *K. ohmeri*, confirming true bloodstream infection. The patient had no conventional risk factors. Antifungal susceptibility testing demonstrated high susceptibility to amphotericin B, voriconazole, and caspofungin, whereas fluconazole showed reduced susceptibility. Due to severe hepatic and renal dysfunction, liposomal amphotericin B was preferred, resulting in rapid clinical improvement and clearance of fungemia. Repeat blood cultures showed no growth, and the patient recovered.

A review of the literature identified only 14 previously reported cases of community-acquired *K. ohmeri* fungemia, most of which occurred in patients with identifiable host risk factors or structural disease. This case presents one of the few reports of primary community-acquired *K. ohmeri* fungemia in an immunocompetent individual and, to our knowledge, shows the first reported association with severe leptospirosis.

*K. ohmeri* should be considered a potential cause of community-acquired fungemia even in immunocompetent hosts. Accurate species identification, repeated blood culture confirmation, and timely antifungal therapy are critical for favourable outcomes. This case expands the clinical spectrum of* K. ohmeri *infection and suggests that severe leptospirosis may be a possible predisposing condition.

## Introduction

*Kodamaea ohmeri*(formerly known as *Pichia ohmeri* or *Yamadazyma ohmeri*) is an emerging opportunistic yeast pathogen in the family Saccharomycetaceae, and the only *Kodamaea* species capable of sustained growth at 37 °C and causing human invasive disease [1]. This pathogen primarily affects immunocompromised individuals. It can cause a wide range of serious infections, including fungemia, peritonitis, endocarditis, and urinary tract infections. It can also lead to pneumonia, keratitis, and skin infections. In severe cases, the mortality rate can reach as high as 50% [2]. Accurate identification poses challenges as conventional phenotypic methods frequently misidentify it as a Candida species, leading to delayed or suboptimal therapy [1]. Systemic infections in immunocompetent subjects without conventional risk factors are rare [3].

Although *K. ohmeri* fungemia has increasingly been reported, community-acquired bloodstream infection in immunocompetent individuals without recognised predisposing factors remains uncommon. To the best of our knowledge, this is the first reported case of *K. ohmeri* fungemia coexisting with severe leptospirosis. We report this case to highlight the occurrence of community-acquired *K. ohmeri* fungemia in a previously healthy individual, the importance of species-level identification, and the challenges of antifungal susceptibility interpretation in the absence of species-specific clinical breakpoints. We also review all published cases of community-acquired *K. ohmeri* fungemia to highlight the uniqueness of this presentation.

## Case presentation

In September 2025, a previously healthy 33-year-old woman with no known comorbidities and no past relevant medical history or exposure presented with a 7-day history of fever, headache, and myalgia. After two days, jaundice appeared, followed by three to four days of altered behavior and incoherent speech. She was initially managed at a local facility from days 5-7 for multi-organ dysfunction syndrome (MODS), pneumonia, shock, acute kidney injury and acute liver injury. Treatment at the facility included intravenous (IV) antibiotics and supplemental oxygen without any central venous lines or invasive procedures. Due to progressive deterioration, she was referred to our tertiary care centre emergency department on day 8 of illness (hospital day 1) and admitted to the medical intensive care unit (MICU) on hospital day 3.

**Figure 1:**
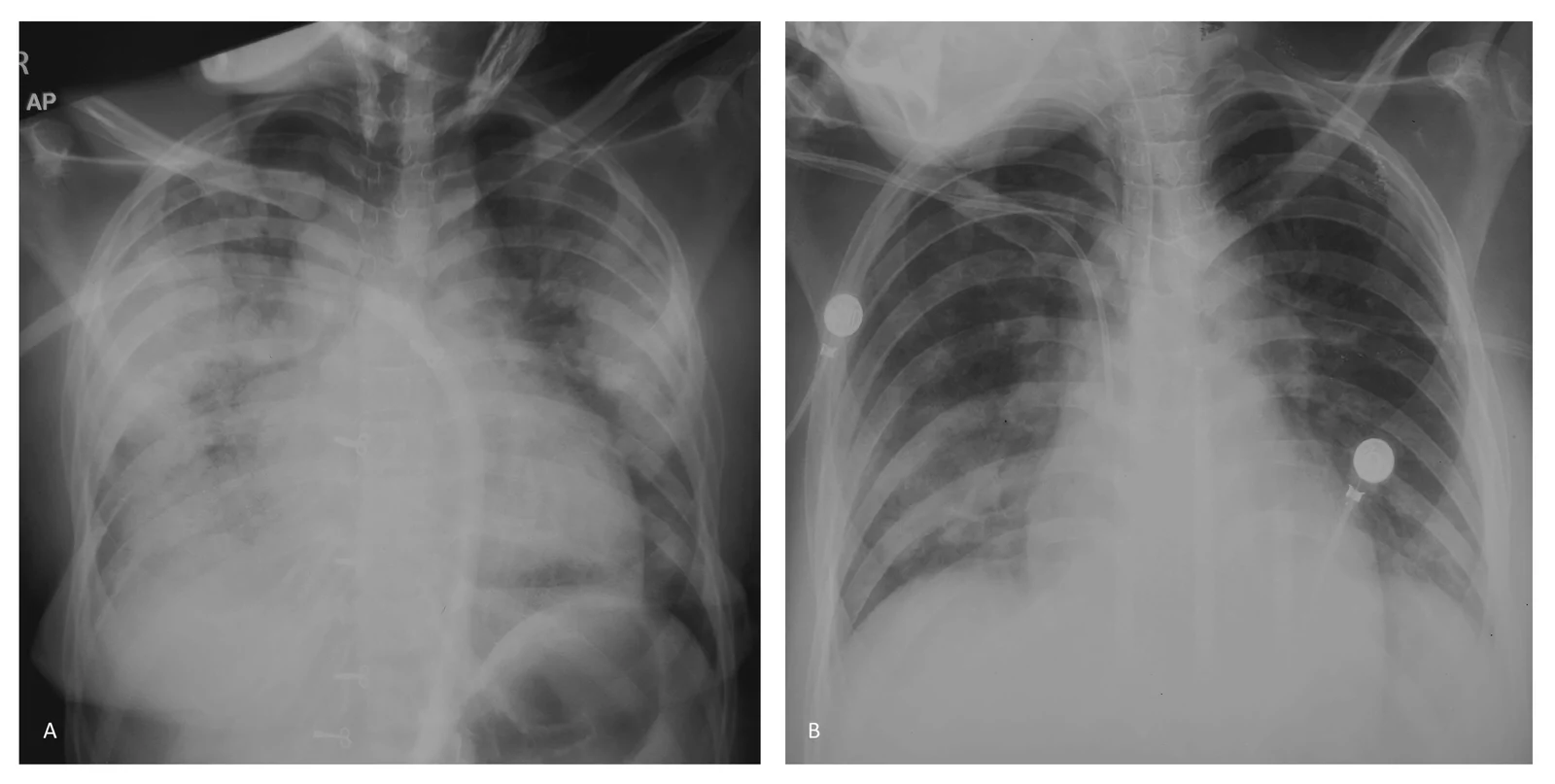
X-ray Chest AP view A: Bilateral fluffy airspace opacities suggestive of acute respiratory distress syndrome visible on day 1 of hospitalisation; B: Significant resolution of opacities visible on day 4 of hospitalisation.

On admission, she required endotracheal intubation and mechanical ventilation for respiratory distress, with norepinephrine infusion support for septic shock. Chest X-ray was suggestive of acute respiratory distress syndrome (ARDS) (Figure 1).

Empirical therapy was initiated with IV meropenem (1 gm, 8-hourly) and doxycycline (100 mg, 12-hourly) for suspected tropical fever and administered for 7 days. Laboratory investigations revealed severe anaemia (haemoglobin: 5.8 g/dL), neutrophilic leukocytosis (total leucocyte count (TLC): 22,180/mm^3^), thrombocytopenia (platelets: 86 × 10^3^/mm^3^), acute kidney injury (urea: 429 mg/dL; creatinine: 10.0 mg/dL), and severe cholestatic hepatitis (total bilirubin: 8.50 mg/dL; direct bilirubin: 8.36 mg/dL; aspartate aminotransferase (AST): 127 U/L; alanine aminotransferase (ALT): 175 U/L). Chest radiography demonstrated infiltrates in the right middle and bilateral lower zones. Transthoracic echocardiography (TTE) was normal with no valvular vegetations. Oliguric acute kidney injury required two sessions of haemodialysis.

**Table 1:** Serial laboratory investigations from admission to recovery Abbreviations: ALT, alanine aminotransferase; AST, aspartate aminotransferase; CRP, C-reactive protein; CRBSI, catheter-related bloodstream infection; MRSA, methicillin-resistant Staphylococcus aureus; TLC, total leucocyte count. Days listed for the blood cultures represent the hospital day on which the blood culture sample was drawn

Parameter (Normal range)	On admission	Post-Extubation (Day 10-12)	MRSA CRBSI/ Endocarditis (Day 28-29)	Pre-Discharge (Day 63)
Haemoglobin (12-15 g/dL)	5.8	7.1	5.7	9.3
TLC (4000-10,000/mm³)	22,180	6,250	20,000	8,060
Platelets (150-450×10³/mm³)	86	180	509	466
Serum Urea (15 – 39 mg/dL)	429	220	33.6	27.8
Serum Creatinine (0.6-1.1 mg/dL)	10.0	7.6	1.4	0.7
Total Bilirubin (0.2 - 1.2 mg/dL)	8.50	2.1	0.85	0.4
Direct Bilirubin (0 - 0.3 mg/dL)	8.36	1.9	0.52	0.3
AST/SGOT (0-45 U/L)	127	62	50.4	53.2
ALT/SGPT (0-45 U/L)	175	67	53.2	87.2
Serum Sodium (135 -145 mEq/L)	135	130	141	139
Serum Potassium (3.5 - 5.5 mEq/L)	5.0	4.3	3.5	4.8
CRP (< 5 mg/L)	42.7	121	154	72.1
Procalcitonin (0.1 ng/mL)	7.96	4.26	—	—
Serum Ferritin (15 – 150 ng/mL)	676	488	—	—
Blood Culture	*K. ohmeri *(Day 2 and 4)	Sterile (Day 11)	MRSA (Day 15, 25, 28)	Negative (Day 42 and 59)

Given her occupational exposure as a paddy-field farmer, a tropical infection screen was performed. On hospital day 4, serological evaluation showed a strongly positive Leptospira IgM antibody (index value 14.989) with J. Mitra Pvt Ltd kits. Paired serological testing was not performed. Scrub typhus IgM ELISA, malaria rapid antigen test (HRP2 and pLDH), and dengue NS1 and IgM ELISA were all negative. Taken together, the compatible clinical syndrome, recent paddy-field exposure, positive Leptospira IgM serology, and a Modified Faine's score of 36 supported a clinical diagnosis of severe leptospirosis [4].

On hospital day 2, blood cultures were taken in the emergency department before inserting a central line. After 48 h of incubation in an automated system (BacT/ALERT Microbial Detection System, bioMérieux SA, France), they turned positive, revealing budding yeast-like cells on day 4. Fluconazole (800 mg loading dose followed by 400 mg once a day) was started empirically, pending species identification and susceptibility data. The isolates were identified as *K. ohmeri* on the VITEK 2 YST card and were confirmed by matrix-assisted laser desorption ionisation-time of flight (MALDI-TOF) mass spectrometry. To exclude contamination, repeat paired blood cultures drawn on hospital day 4 yielded *K. ohmeri* in two of four bottles, confirming true bloodstream infection. Antifungal susceptibility testing via the VITEK 2 YS08 card demonstrated active-range minimum inhibitory concentrations (MICs) for all drugs based on surrogate Candida criteria [5]. The specific minimum inhibitory concentration (MIC) values were voriconazole <0.12 µg/mL, amphotericin B 0.5 µg/mL, and caspofungin <0.12 µg/mL; however, the fluconazole MIC was 8 µg/mL, which is relatively elevated compared to most Candida species and indicates reduced susceptibility.

Given that the admission blood culture showed growth of *K. ohmeri*, no central venous catheters were placed at the referring facility, peripheral IV sites showed no phlebitis, and the patient lacked traditional risk factors for invasive candidiasis or opportunistic fungemia (such as diabetes, malignancy, neutropenia, corticosteroid therapy, total parenteral nutrition, or pre-existing central venous access), the case was designated as a primary, community-acquired *K. ohmeri* fungemia presenting simultaneously with severe leptospirosis. Fluconazole was discontinued after species identification. Although the isolate had a low voriconazole MIC, severe cholestatic hepatitis and dialysis-dependent acute kidney injury favoured treatment with liposomal amphotericin B (L-AmB). L-AmB was initiated at 5 mg/kg/day on hospital day 7, based on its predominantly non-renal elimination and pharmacokinetic advantages in haemodialysis patients.

**Figure 2:**
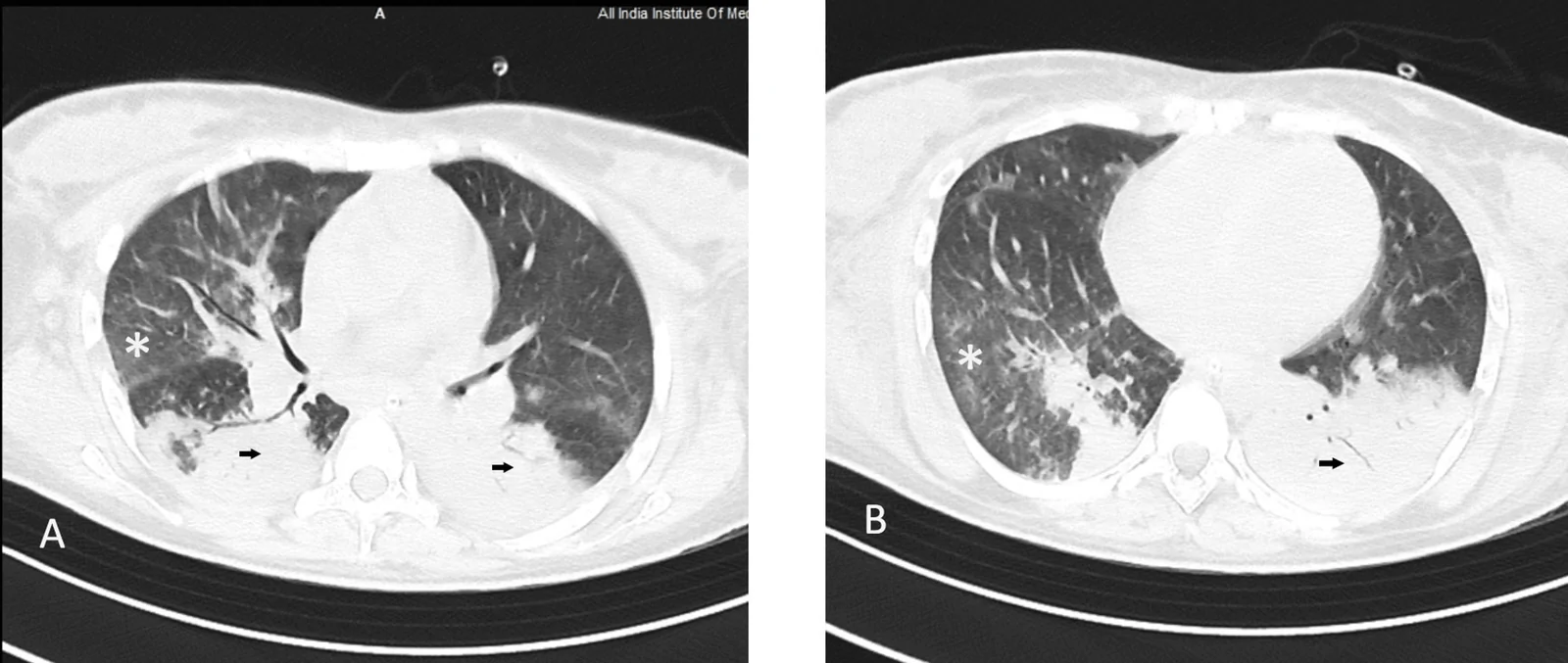
HRCT chest High-resolution computed tomography (HRCT) chest showing ground-glass opacities (asterisk) in the right upper and middle lobes, with areas of consolidation in the bilateral lower lobes (black arrows).

A repeat transthoracic echocardiography was performed after the second positive blood culture report showed no vegetations. Secondary workup, including human immunodeficiency virus (HIV), hepatitis B surface antigen, and hepatitis C virus serology, was negative, and her absolute CD4 count was normal at 1488 cells/mm^3^. Fundoscopy was normal. High-resolution computed tomography (HRCT) chest demonstrated bilateral lower-lobe consolidation with ground-glass opacities (Figure 2). An endotracheal aspirate culture grew *Acinetobacter baumannii*, which was considered a coloniser, as hypoxaemia was improving. The shock resolved, renal function progressively improved, the fever subsided, and the patient was extubated after six days. Blood cultures repeated on hospital day 11 confirmed clearance of the bloodstream infection. She was transferred to the ward on hospital day 12 with a central venous catheter (CVC) for ongoing venous access; L-AmB was continued for a total of 18 days (two weeks after the first negative culture).

**Figure 3:**
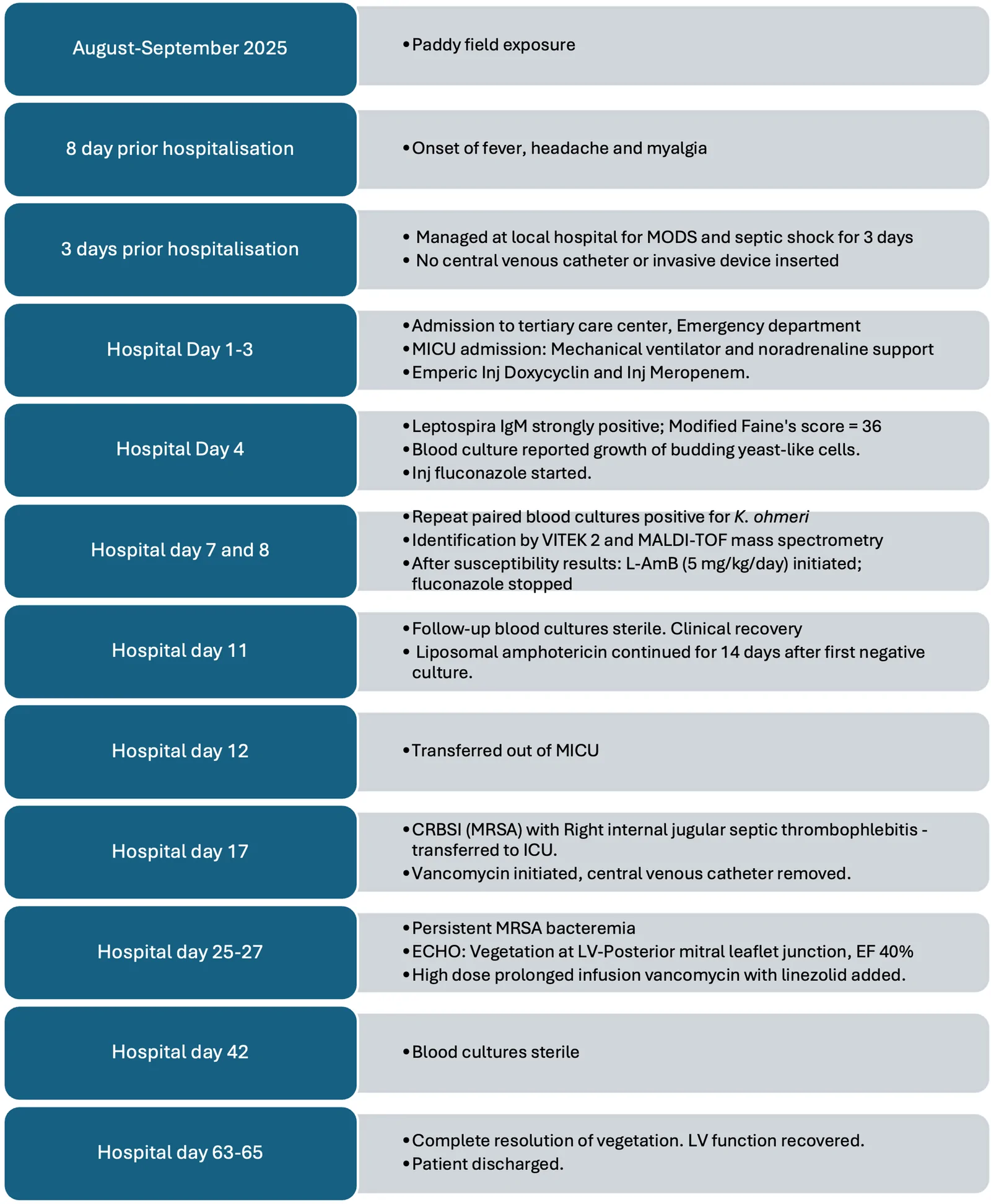
Clinical timeline of events MICU: Medical Intensive Care Unit; IgM: Immunoglobulin M; MODS: Multi-Organ Dysfunction Syndrome; L-AmB: Liposomal Amphotericin B; MRSA: Methicillin-Resistant Staphylococcus aureus; CRBSI: Catheter Related Bloodstream Infection; LV: Left Ventricle; EF: Ejection Fraction; Inj: Injection; ECHO: Echocardiogram

On hospital day 15, she developed recurrent fever, and paired blood cultures were obtained. On hospital day 17, blood cultures grew methicillin-resistant *Staphylococcus aureus* (MRSA), consistent with catheter-related bloodstream infection (CRBSI). This was complicated by right internal jugular septic thrombophlebitis, which further led to mitral valve infective endocarditis on hospital day 25. The bacteremia persisted despite CVC removal and initial vancomycin therapy (750 mg, 8-hourly) guided by susceptibility testing. Vancomycin was subsequently escalated to 1 g every 8 hours administered as a prolonged infusion, with addition of linezolid (600 mg 12-hourly). Following this, blood cultures became sterile and treatment was continued (total 46 days) until hospital day 63 (Figure 3). Thrombosis and vegetation resolved completely on follow-up imaging on hospital day 63. The patient was ultimately discharged with fully normalised cardiac and renal function. Table 1 summarises serial laboratory investigations, and Figure 3 shows the timeline of clinical events.

## Discussion

We searched the PubMed, Embase, Web of Science, and CNKI (China National Knowledge Infrastructure) databases for relevant literature published up to June 2026 using the following keywords: (*Kodamaea* OR *Pichia* OR *Yamadazyma*) AND *ohmeri*. We identified 79 studies involving 107 patients, including 4 nosocomial outbreaks. Of these, we identified 14 cases of community-acquired *K. ohmeri* fungemia [3,6-18] (summarised in Table 2). We defined community-acquired fungemia as a bloodstream infection present at admission or identified from blood cultures obtained before significant healthcare exposure or placement of intravascular devices, in the absence of recognised healthcare-associated risk factors. This case is unique in two ways: it is a rare instance of primary, community-acquired *K. ohmeri* fungemia in a patient without pre-existing immunocompromising conditions with no conventional risk factors, and it is the first documented case to co-occur with severe leptospirosis.

**Table 2:** Community-acquired Kodamaea ohmeri fungemia cases reported in the literature AmB: amphotericin B deoxycholate; L-AmB: Liposomal amphotericin B; ANID: anidulafungin; BAL: bronchoalveolar lavage; Caspo: caspofungin; FCZ: fluconazole; HLH: haemophagocytic lymphohistiocytosis; ICZ: itraconazole; ISA: isavuconazole; IV: intravenous; L-AmB: liposomal amphotericin B; Mica: micafungin; VCZ: voriconazole.

Author, Year	Country	Age (y)/Sex	Infection Type	Underlying Disease	Risk Factors	Isolation Source	Treatment	Outcome
Present case, 2025	India	33/F	Leptospirosis +* K. ohmeri *fungaemia	None	Severe leptospirosis	Blood	L-AmB	Recovered
Chen et al., 2026 [6]	Taiwan	58/M	*K. ohmeri* endocarditis	None	Not stated	Blood, vegetation	L-AmB, VCZ	Recovered
Sun et al., 2024 [7]	China	66/M	*K. ohmeri* urosepsis with fungaemia	Obstructive uropathy	Not stated	Blood, urine	FCZ	Recovered
Wilcock et al., 2023 [8]	USA	40/F	*K. ohmeri* endocarditis	None	IV drug use	Blood, valve tissue	Mica, AmB, ICZ, Caspo	Recovered
Li et al., 2022 [9]	China	37/M	Pulmonary aspergillosis + Enterogenous *K. ohmeri *with fungaemia	None	Not stated	Stool, blood	Caspo	Recovered
Wang et al., 2022 [10]	China	52/M	*K. ohmeri* necrotising cellulitis + fungaemia	AIDS (CD4 132/mm³)	Skin breakdown	Wound, blood	FCZ, AmB	Recovered
Al-Salameh et al., 2020 [11]	USA	35/M	*K. ohmeri* endocarditis	Prolonged QT, chronic hepatitis C	IV drug use	Blood	ISA	Recovered
Diallo et al., 2019 [3]	France	81/F	*K. ohmeri* pneumonia + fungaemia	Cognitive disorder	Not stated	BAL, blood	VCZ	Recovered
Huang et al., 2018 [12]	China	63/M	*K. ohmeri* fungaemia	Metastatic gastric adenocarcinoma	Not stated	Blood	FCZ, ANID	Died
Ni et al., 2018 [13]	China	57/M	*K. ohmeri* endocarditis + HLH	None	Not stated	Blood	VCZ	Recovered
Cao et al., 2016 [14]	China	69/F	*K. ohmeri* endocarditis	Asthma, rheumatic heart disease	Steroids	Blood	VCZ	Died
Yanghua et al., 2010 [15]	China	43/M	*K. ohmeri* endocarditis	Rheumatic heart disease, hepatitis B	Not stated	Blood	ICZ	Recovered
Yang et al., 2009 [16]	China	71/M	Cellulitis + *K. ohmeri* fungaemia	Diabetes mellitus	Not stated	Blood	FCZ, AmB	Recovered
João et al., 2002 [17]	Portugal	42/M	*K. ohmeri* endocarditis	Chronic hepatitis C, peripheral vasculopathy	IV drug use, skin lesions	Blood, embolus	L-AmB	Recovered
Reina et al., 2002 [18]	USA	76/M	*K. ohmeri* endocarditis	None	Bioprosthetic mitral valve	Blood	FCZ, AmB	Recovered

Analysis of previously reported community-acquired cases showed a predominance among older adults (median age 57.5 years) and males (11/14 cases). Endocarditis was the most frequent presentation (8/14 cases), often associated with predisposing valvular abnormalities or intravenous drug use. Ten cases presented with primary bloodstream infections, while four were secondary (due to pneumonia, obstructive urosepsis, gut infection, and necrotising cellulitis). In contrast to the high mortality rates associated with ICU outbreaks, the overall mortality in community-acquired cohorts was significantly lower at 14.3% (2/14), with deaths occurring primarily in settings of delayed diagnosis or advanced haematological malignancy.

Among these community-acquired cases, the majority had identifiable host risk factors such as diabetes mellitus, HIV infection, metastatic malignancy, corticosteroid exposure, structural heart disease, obstructive uropathy, chronic hepatitis or intravenous drug use. Only four of these cases had no recognisable host risk factor [3,6,9,13]. Our patient was previously healthy and immunocompetent (CD4 count 1,488 cells/mm^3^; HIV-negative, HBsAg-negative, HCV-negative) and had no invasive devices before the onset of illness. The pre-device timing of positive blood cultures and the confirmed repeat growth exclude healthcare acquisition and contamination. These findings support community-acquired over healthcare-associated fungemia.

The portal of entry of *K. ohmeri* in this patient remains uncertain. However, her occupation as a paddy-field farmer involved frequent exposure to soil, stagnant water, and decaying vegetation, which could have exposed her to opportunistic yeasts. In addition, severe leptospirosis is associated with cytokine storm and subsequent immunoparalysis, leading to sepsis and associated organ failures [19]. Endothelial injury, immune dysregulation, and multiorgan dysfunction might increase vulnerability to bloodstream invasion by *K. ohmeri*. However, a direct causal link cannot be confirmed based on a single case. Additionally, gastrointestinal translocation during severe sepsis and multiorgan failure is another plausible mechanism. Although the cause cannot be established, environmental exposure combined with host barrier disruption may have contributed to *K. ohmeri* fungemia.

Antifungal susceptibility interpretation for *K. ohmeri* remains challenging because neither Clinical and Laboratory Standards Institute (CLSI) nor European Committee on Antimicrobial Susceptibility Testing (EUCAST) has established species-specific clinical breakpoints or epidemiological cutoff values. MIC values must therefore be interpreted in the context of published susceptibility distributions and clinical outcomes. While our centre relied on automated VITEK 2 cards, reference CLSI broth microdilution testing was unavailable, which represents a limitation of the present report. In our isolate, the susceptibility profile (low MICs to amphotericin B, voriconazole, and caspofungin with reduced susceptibility to fluconazole) is consistent with previously reported patterns [20]. L-AmB was selected over voriconazole because of the patient's critical illness, extensive published experience with amphotericin B for invasive yeast infections, and its fungicidal activity, together with the hepatotoxicity risk and unpredictable pharmacokinetics of azoles in the context of severe cholestatic hepatitis and dialysis-dependent acute kidney injury (AKI). Rapid culture clearance and clinical improvement following L-AmB supported the appropriateness of this choice.

This report has a few limitations. First, confirmatory testing for leptospirosis, including PCR and paired serology or microscopic agglutination testing, was not available; therefore, the diagnosis of leptospirosis was based on the compatible clinical and epidemiological presentation, positive Leptospira IgM serology, and Modified Faine's score [4]. Second, CLSI and EUCAST have not established species-specific clinical breakpoints or epidemiological cutoff values for *K. ohmeri*, limiting definitive interpretation of the antifungal MICs. Third, as this is a single case, a causal association between severe leptospirosis and susceptibility to *K. ohmeri* bloodstream infection cannot be established.

## Conclusions

Community-acquired *K. ohmeri* fungemia can occur in an immunocompetent individual without conventional predisposing factors. Early species identification is important for appropriate management. In the absence of species-specific antifungal breakpoints, it is important to interpret MIC results cautiously in conjunction with the clinical context. To our knowledge, this is the first reported case of K. ohmeri fungemia occurring concurrently with a clinical diagnosis of severe leptospirosis. It remains unclear whether severe leptospirosis served as a predisposing factor for K. ohmeri fungemia.
